# Obesity Biomarkers: Exploring Factors, Ramification, Machine Learning, and AI‐Unveiling Insights in Health Research

**DOI:** 10.1002/mco2.70169

**Published:** 2025-06-22

**Authors:** Ankita Awari, Deepika Kaushik, Ashwani Kumar, Emel Oz, Kenan Çadırcı, Charles Brennan, Charalampos Proestos, Mukul Kumar, Fatih Oz

**Affiliations:** ^1^ Department of Food Technology and Nutrition Lovely Professional University Phagwara Punjab India; ^2^ Department of Biotechnology Faculty of Applied Sciences and Biotechnology Shoolini University Solan Himachal Pradesh India; ^3^ Institute of Food Technology Bundelkhand University Jhansi India; ^4^ Department of Food Engineering Faculty of Agriculture Ataturk University Erzurum Turkey; ^5^ Department of Internal Medicine Erzurum Regional Training and Research Hospital Health Sciences University Erzurum Turkey; ^6^ School of Science RMIT University Melbourne Victoria Australia; ^7^ Laboratory of Food Chemistry Department of Chemistry School of Sciences National and Kapodistrian University of Athens Zografou Athens Greece; ^8^ East Anatolian High Technology Research and Application Center (DAYTAM) Ataturk University Erzurum Turkey; ^9^ Department of Food Engineering Kyrgyz‐Turkish Manas University, Engineering Faculty Bishkek Kyrgyzstan

**Keywords:** biomarker, data mining, knowledge discovery in databases (KDD), obesity, omic biomarker, oxidative stress biomarker

## Abstract

Biomarkers play a pivotal role in the detection and management of diseases, including obesity—a growing global health crisis with complex biological underpinnings. The multifaceted nature of obesity, coupled with socioeconomic disparities, underscores the urgent need for precise diagnostic and therapeutic approaches. Recent advances in biosciences, including next‐generation sequencing, multi‐omics analysis, high‐resolution imaging, and smart sensors, have revolutionized data generation. However, effectively leveraging these data‐rich technologies to identify and validate obesity‐related biomarkers remains a significant challenge. This review bridges this gap by highlighting the potential of machine learning (ML) in obesity research. Specifically, it explores how ML techniques can process complex data sets to enhance the discovery and validation of biomarkers. Additionally, it examines the integration of advanced technologies for understanding obesity mechanisms, assessing risk factors, and optimizing treatment strategies. A detailed discussion is provided on the applications of ML in multi‐omics analysis and high‐throughput data integration for actionable insights. The academic value of this review lies in synthesizing the latest technological and analytical innovations in obesity research. By providing a comprehensive overview, it aims to guide future studies and foster the development of targeted, data‐driven strategies in obesity management.

## Graphical abstract

The graphical abstract emphasizes the complex nature of obesity, focusing on the interaction of behavioral, sociodemographic, genetic, and biomarker factors. It illustrates how lifestyle habits, demographics, and genetic predispositions influence obesity risk. Various biomarkers are used to detect and diagnose obesity, including diagnostic, safety, pharmacodynamic, monitoring, predictive, and susceptibility types. Advanced AI and programs analyze these biomarkers to provide personalized recommendations. These insights help individuals manage and overcome obesity by suggesting tailored lifestyle changes and medications, thus optimizing treatment and improving health outcomes.

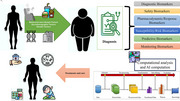

## 1 Introduction

Obesity has escalated into a global public health crisis, affecting diverse populations and exacerbating health disparities among vulnerable and disadvantaged groups [1]. Characterized by excessive and abnormal body fat accumulation, obesity leads to physiological, anatomical, and functional impairments across all age groups [2]. Since 1975, obesity prevalence has nearly tripled, with approximately 650 million adults, 340 million adolescents, and 39 million children affected globally as of 2022 (WHO, 2022). In India, obesity rates have increased from 21% to 24% in women and 19% to 23% in men, affecting around 135 million individuals (NFHS, 2019–20). However, recent epidemiological studies indicate that these numbers are continuing to rise, necessitating urgent and effective intervention strategies.

Despite increased awareness, the pathophysiology of obesity remains complex, involving hormonal, metabolic, neurochemical, immune‐inflammatory abnormalities, and gene–environment interactions [3]. Several studies have explored obesity's link to comorbidities such as type 2 diabetes, nonalcoholic fatty liver disease (NAFLD), cardiovascular diseases, and certain cancers, yet population‐level interventions remain limited [3]. Advances in biosciences, including multi‐omics analysis and machine learning (ML), have introduced new opportunities for identifying obesity‐related biomarkers, but integrating these findings into clinical practice remains a challenge [4].

This review aims to bridge these gaps by exploring the latest advancements in obesity research, particularly the role of ML in biomarker discovery. It provides a comprehensive synthesis of existing studies, highlights emerging trends, and discusses challenges in translating research into effective clinical and public health strategies.The review is structured as follows: First, it presents an overview of obesity's epidemiology, pathophysiology, and associated risk factors. Next, it examines the role of advanced technologies, including multi‐omics and ML, in understanding obesity mechanisms. The final sections focus on the integration of high‐throughput data, research gaps, and future directions in obesity prevention and management. By addressing these aspects, this review aims to provide valuable insights for researchers and healthcare professionals working toward data‐driven obesity solutions.

## 2 Factors That Contribute to the Likelihood of Obesity

Obesity is a multifactorial condition influenced by a complex interplay of genetic, environmental, metabolic, endocrine, and gut‐related factors, as summarized in Table 1. Genetic predispositions can increase susceptibility to weight gain, especially when coupled with environmental factors such as sedentary lifestyles and high‐calorie diets. Metabolic factors, including impaired energy regulation and lipid metabolism, further exacerbate weight accumulation. Endocrine imbalances, such as insulin resistance and hormonal dysregulation, along with immune system alterations, contribute to chronic inflammation associated with obesity. Additionally, gut microbiota composition plays a pivotal role, influencing nutrient absorption, appetite regulation, and systemic metabolism.

**TABLE 1:** Predisposing factors contributing to the likelihood for obesity.

Category	Factors/implications	References
Sociodemographic factors	‐ Socioeconomic status: Low income, urban residency, private school attendance. ‐ Transition from rural to urban living. ‐ Early undernutrition and later obesity.	[5, 6, 7, 8, 9, 10, 11]
Behavioral factors	‐ Calorie‐dense foods: Sweets, sugars, fats, alcohol. ‐ Refined carbohydrates, ultra‐processed foods. ‐ Monotonous diets, eating behaviors. ‐ Stress, sedentary lifestyle.	[11, 12, 13, 14, 15, 16, 17, 18]
Genetic factors	‐ Genetic predisposition with over 250 genes linked to obesity. ‐ FTO gene's role in obesity and type 2 diabetes. ‐ Cross‐sectional studies evaluating gene‐obesity links.	[8, 15]
Health ramifications	‐ Metabolic disorders: Diabetes, dyslipidemia, hypertension. ‐ Cardiovascular disease, cancer, obstructive sleep apnea. ‐ Fertility issues, Barrett's esophagus.	[19, 20, 21, 22, 23, 24, 25, 26, 27]

## 3 Biomarkers

A biomarker is defined as “a characteristic measured as an indicator of normal biological processes, pathogenic processes, or responses to an exposure or intervention” (FDA‐NIH Biomarker Working Group, 2016). This broad definition encompasses histologic, molecular, physiological, and radiographic aspects, making biomarkers crucial in research, medical product development, and healthcare. Unlike clinical outcome assessments (COAs), which directly measure patient‐relevant outcomes such as feelings, behaviors, or survival, biomarkers predict COAs but do not directly assess clinical outcomes. Emphasizing the need for long‐term follow‐up and validation studies is crucial to establish the reliability, reproducibility, and clinical utility of obesity biomarkers. These studies ensure that biomarkers consistently predict clinical outcomes across diverse populations and over time, thereby reinforcing their relevance in personalized treatment strategies. Additionally, they provide the robust evidence necessary for integrating biomarkers into routine clinical practice, ultimately improving patient care and advancing obesity management. Emphasizing the need for long‐term follow‐up and validation studies is crucial to establish the reliability, reproducibility, and clinical utility of obesity biomarkers. These studies ensure that biomarkers consistently predict clinical outcomes across diverse populations and over time, thereby reinforcing their relevance in personalized treatment strategies. Additionally, they provide the robust evidence necessary for integrating biomarkers into routine clinical practice, ultimately improving patient care and advancing obesity management. Once validated, biomarkers can serve as primary endpoints for regulatory approval, particularly when effective treatments are not available. They also enable expedited approval pathways as outlined by regulatory bodies like the FDA. In clinical studies, both biomarkers and COAs require rigorous scientific methods, with endpoints defined as specific variables for statistical analysis to address research questions (FDA‐NIH Biomarker Working Group, 2016). To enhance discussions on clinical applications, it is important to focus on biomarkers used for early detection of obesity‐related complications. For instance, HbA1c is a key biomarker for diabetes, demonstrating high diagnostic accuracy with a sensitivity of 68% and specificity of 88% [28]. C‐reactive protein (CRP) is used to assess cardiovascular risk, with sensitivity ranging from 60%–80% and specificity from 50%–70% [29]. Lipid profiles are valuable for cardiovascular diseases, showing high sensitivity but variable specificity depending on additional risk factors [30]. Biomarkers play a pivotal role in the detection and management of diseases, including obesity—a growing global health crisis with complex biological underpinnings. The multifaceted nature of obesity, coupled with socioeconomic disparities, underscores the urgent need for precise diagnostic and therapeutic approaches. Recent advances in biosciences, including next‐generation sequencing (NGS), multi‐omics analysis, high‐resolution imaging, and smart sensors, have revolutionized data generation. However, the effective utilization of these data‐rich technologies to identify and validate obesity‐related biomarkers remains a significant challenge.

This review was undertaken to address this critical challenge and highlight the untapped potential of advanced analytical techniques in obesity research. It explores how ML can process complex data sets to enhance biomarker discovery and validation. Additionally, it examines the integration of advanced technologies for understanding obesity mechanisms, assessing risk factors, and optimizing treatment strategies. A detailed discussion is provided on the applications of ML in multi‐omics analysis and high‐throughput data integration for actionable insights.

The academic value of this review lies in synthesizing the latest technological and analytical innovations, offering guidance for future studies and fostering the development of targeted, data‐driven strategies in obesity management.

## 4 Establishing Biomarkers: From Correlation to Valid Surrogacy

A common misconception in biomarker assessment is assuming that a correlation between a biomarker level and a clinical outcome signifies the biomarker's reliability as a surrogate. For a biomarker to qualify as a surrogate, it must explain changes in the clinical outcome, not merely correlate with it. This explanation requires statistical inference from observations across various treatments affecting the biomarker differently. Consequently, most biomarkers lack validity as surrogates, and even validated surrogates are specific to particular ailments.

**FIGURE 1:**
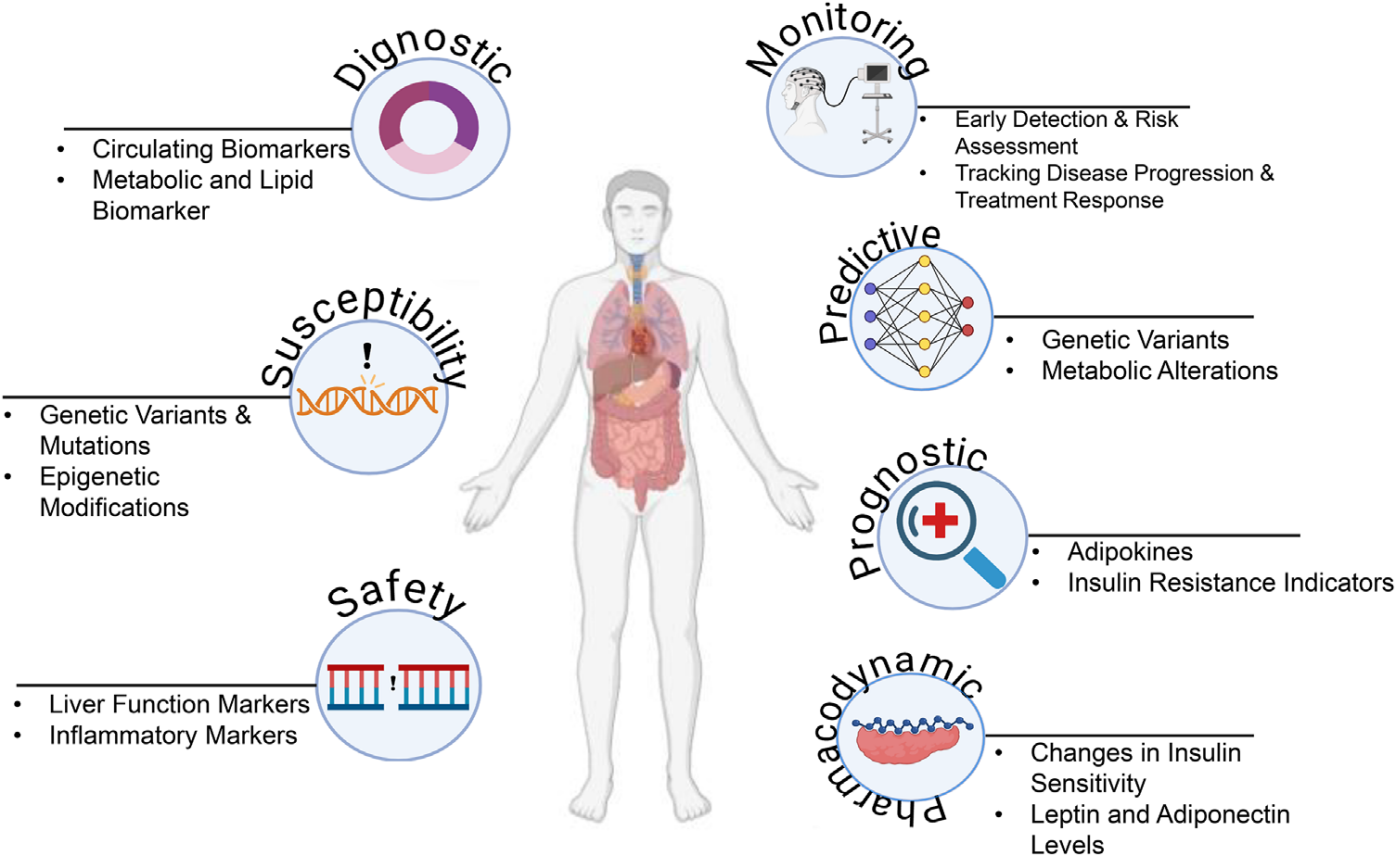
Types of biomarkers. This figure categorizes biomarkers into diagnostic, susceptibility, safety, monitoring, predictive, prognostic, and pharmacodynamic types. It highlights their roles, such as disease detection, genetic predisposition, treatment response monitoring, and metabolic regulation.

Fleming, DeMets, and Prentice's seminal work (Figure 1) clarifies that “a correlation does not a surrogate make” due to the complexity of therapeutic actions and biological mechanisms. Treatments may alter clinical outcomes without affecting the surrogate, or vice versa, as seen with HDL cholesterol levels and atherosclerosis. Thus, surrogates must be validated through extensive research and multiple clinical trials to ensure their relevance across various treatments before regulatory approval by the FDA [31].

## 5 Classification and Applications of Biomarkers

### 5.1 Diagnostic Biomarker

Diagnostic biomarkers are used to identify specific disease subtypes or confirm the presence of a particular condition. With the advancement of precision medicine, these biomarkers are shifting from traditional organ‐based classifications to more molecular and imaging‐based approaches. This transition is evident in cancer diagnostics where biomarkers now facilitate detailed reclassification of the disease. However, their application must be carefully evaluated, as their utility can vary significantly between broad scientific concepts and specific clinical contexts (FDA‐NIH Biomarker Working Group, 2016) [32, 33]

### 5.2 Monitoring Biomarker

Monitoring biomarkers are crucial for evaluating the progression of a disease, assessing exposure to substances, and tracking the effects of treatments. They are integral in clinical care: For instance, LDL cholesterol levels are used to manage hyperlipidemia, while CD4 counts are essential in monitoring HIV treatment efficacy. Despite their importance, defining precise changes in these biomarkers that necessitate clinical intervention can be challenging. Monitoring biomarkers also play a role in ensuring patient safety during drug development, with repeated measurements used to detect potential adverse effects [34].

### 5.3 Pharmacodynamic/Response Biomarkers

Pharmacodynamic or response biomarkers change in response to drug administration or environmental exposure. They are crucial in the early stages of drug development for determining appropriate dosing and assessing therapeutic responses. However, variability in patient responses can complicate the validation of these biomarkers. Ensuring that observed changes are indicative of the expected treatment response requires rigorous validation [35].

In oncology, biomarkers such as CA‐125 are used to monitor treatment response in ovarian cancer, allowing for the refinement of therapeutic approaches based on biomarker levels [36]. Similarly, in diabetes management, biomarkers like HbA1c are employed to evaluate glucose control and guide adjustments in insulin therapy [37]. These examples illustrate the role of response biomarkers in adjusting therapeutic strategies and predicting patient outcomes.

### 5.4 Predictive Biomarker

Predictive biomarkers forecast the likelihood of a positive or negative response to a treatment or environmental exposure based on the presence or change in the biomarker. These biomarkers are crucial in the design and implementation of clinical trials, allowing for more targeted therapies. For example, LDL cholesterol serves as a predictive biomarker for atherosclerosis and related cardiovascular events. By focusing on individuals with elevated levels, predictive biomarkers enhance the precision of therapeutic interventions [38, 39]

### 5.5 Safety Biomarker

Safety biomarkers are used to monitor organ function and potential damage during drug development. Tests for liver function (e.g., transaminases) and kidney function (e.g., serum creatinine) are well established. Despite their utility, the need for biomarkers with higher specificity and sensitivity for detecting organ toxicity remains critical. Recent efforts, such as those by the Critical Path Institute's Predictive Safety Testing Consortium, aim to validate new biomarkers that can more accurately detect drug‐induced organ damage [39, 40].

### 5.6 Susceptibility/Risk Biomarker

**FIGURE 2:**
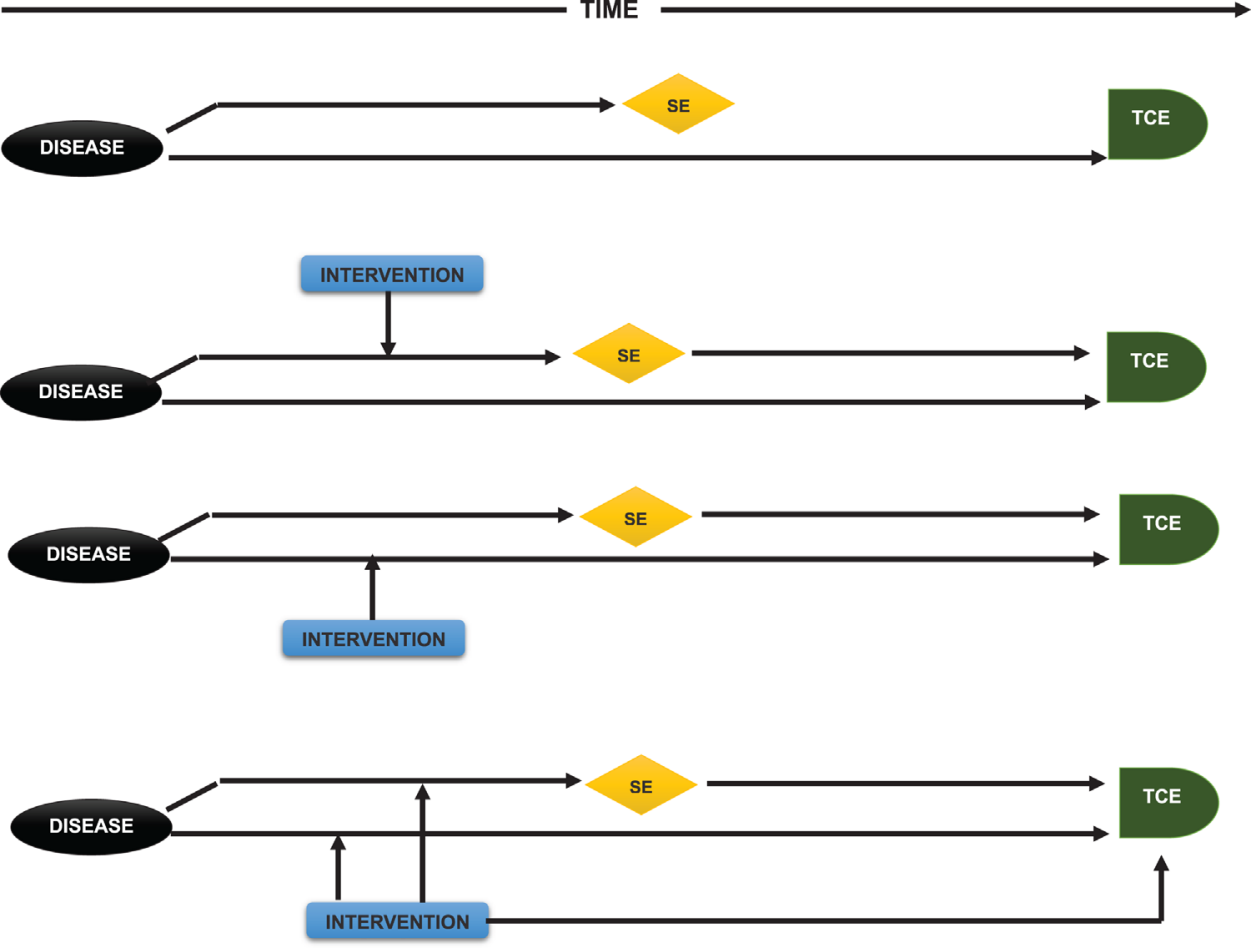
Causes of surrogate endpoints failure. (A) In this scenario, the condition affects the purported surrogate endpoint and the actual clinical outcome differently, rendering any association between the two invalid. (B) The hypothesized surrogate endpoint is influenced by the intervention, which partly affects the actual clinical outcome. However, the disease also influences the actual clinical outcome through other processes, making the change in the purported surrogate a poor indicator of the alteration in the actual clinical outcome. (C) The hypothesized surrogate endpoint is impacted by the intervention through mechanisms unrelated to those affecting the actual clinical outcome. Therefore, it is challenging to accurately determine whether the change in the surrogate endpoint represents a change in the actual clinical outcome. (D) All the aforementioned issues are present and accounted for.

Susceptibility or risk biomarkers predict the likelihood of developing a disease in individuals who do not yet exhibit symptoms. These biomarkers are essential for epidemiological studies focused on disease onset and prevention. They provide insights into the risk of developing specific conditions and are akin to prognostic biomarkers in their focus on early disease prediction rather than post‐diagnosis prognosis (FDA‐NIH Biomarker Working Group, 2020). The different biomarkers are depicted in Figure 2.

## 6 Obesity‐Specific Biomarkers: Unraveling Their Role and Clinical Implication

### 6.1 MicroRNA (miRNA)

miRNAs are increasingly recognized for their role in obesity and associated metabolic disorders. In severely obese adolescents, miRNAs have shown significant correlations with adipokines such as adiponectin and leptin, as well as with leptin/adiponectin (L/A) ratios. Elevated expression of miRNAs like miR‐222, miR‐142‐3p, miR‐140‐5p, and miR‐143, alongside miR‐122 and miR‐34a, is associated with obesity, NAFLD, and insulin resistance. These circulating miRNAs (cmiRNAs) hold promise as diagnostic markers for cardiovascular and other pathological conditions [41]. The regulation of adipogenesis by miRNAs was first identified in Drosophila, with miR‐14 and miR‐278 suppressing fat metabolism by targeting p38 and MAPK [42]. In mammals, various miRNAs influence glucose and lipid metabolism, impacting adipocyte differentiation and insulin signaling. For example, miR‐143 and miR‐145 act as positive regulators of adipocyte differentiation through ERK5 signaling [43]. However, their elevated expression in obesity can disrupt glucose homeostasis by targeting ORP8, thus inhibiting the PI3K–AKT pathway [42, 43]. Conversely, miR‐27a and miR‐130a suppress adipocyte differentiation by targeting PPARγ, a key transcription factor in adipogenesis. Overexpression of miR‐27 impedes adipocyte formation and reduces PPARγ and Cebpa expression, highlighting its role in counteracting adipogenesis [44]. miR‐124 promotes adipogenesis by targeting Dlx5, a pro‐osteogenic factor [45]. Let‐7 regulates glucose metabolism and insulin resistance by targeting IGF1R, INSR, and IRS2, and modulates adipogenesis through HMGA2 expression [46]. miR‐206 inhibits LXRα, affecting lipid metabolism [47]. Additionally, miR‐26b increases adipogenic markers and lipid accumulation in cells by suppressing PTEN [48]. miR‐375 and other miRNAs such as hsa‐miR‐130b‐3p and hsa‐miR‐142‐5p are linked to obesity and insulin secretion modulation [49, 50]. Sirtuins (SIRTs) interact with miRNAs to regulate adipogenesis. SIRT1, a nuclear deacetylase, inhibits adipogenesis by interacting with NCoR and SMRT, and modulates the expression of brown and white adipose tissue genes. SIRT2 suppresses adipogenesis by deacetylating FOXO1 and inhibiting PPARγ, while SIRT7 is essential for adipocyte differentiation. Targeting specific SIRTs may offer new therapeutic strategies for obesity [51]. Early detection of cmiRNAs and their roles in cholesterol and lipid metabolism (e.g., miR‐33‐3p and miR‐223) provides valuable insights for diagnosing and managing obesity‐related metabolic disorders.

### 6.2 Inflammatory Biomarkers

Obesity is strongly correlated with inflammatory biomarkers, such as CRP, interleukin‐6 (IL‐6), and tumor necrosis factor‐alpha (TNF‐α), both IL‐6 and TNF‐α are produced by adipocytes and act as adipokines and inflammatory markers. Elevated levels of these cytokines are linked to increased body fat, particularly abdominal fat, and insulin resistance. Subcutaneous adipose tissue, especially in abdominal obesity, releases IL‐6 at higher levels, and genetic variability in the IL‐6 gene is associated with adiposity [52, 53]. However, in metabolically healthy obese (MHO) individuals, inflammation is lower, challenging the use of these biomarkers for predicting metabolic health. Further research should focus on understanding the stability of the MHO phenotype and its impact on inflammatory biomarker profiles over time [54]. Biomarkers such as leptin, adiponectin, and certain inflammatory cytokines (e.g., IL‐6 and TNF‐α) have shown promise in predicting the risk of obesity and its associated complications. For instance, elevated levels of leptin or low levels of adiponectin in pre‐obese individuals may indicate a higher risk for developing obesity‐related metabolic disorders. By monitoring these biomarkers, healthcare providers can identify at‐risk individuals early and implement targeted lifestyle changes or preventive measures [52]

### 6.3 Adipocytokines

Adipocytokines play a crucial role in linking obesity with associated health issues. Among them, TNF‐α and IL‐6 serve dual roles as both inflammatory markers and adipokines, contributing to the complex relationship between inflammation, metabolic dysfunction, and obesity. These cytokines are involved in various metabolic processes, and their elevated expression in adipocytes can exacerbate obesity‐related complications. Plasminogen Activator Inhibitor‐1 (PAI‐1) is a notable adipocytokine contributing to metabolic problems related to obesity, though its mechanisms require further study. Other biomarkers such as adiponectin, omentin, apelin, leptin, resistin, and fatty‐acid‐binding protein‐4 are also under investigation for their relevance to obesity.

TNF‐alpha is a key cytokine influencing various metabolic processes. It raises plasma triglycerides and very low‐density lipoprotein (VLDL) levels and stimulates lipolysis in adipocytes. Elevated TNF‐alpha expression in adipocytes of obese individuals correlates with obesity and hyperinsulinemia. However, TNF‐alpha blockade has not consistently improved insulin sensitivity in humans. The mechanism by which TNF‐alpha induces insulin resistance is not fully understood, but one proposed pathway involves the activation of the JNK pathway, leading to serine 307 phosphorylation of insulin receptor substrate‐1 (IRS‐1). This disrupts insulin signaling by interfering with IRS‐1 and insulin receptor interaction and reduces the transcriptional activity and expression of insulin signaling molecules, including IRS‐1 and GLUT‐4. Additionally, TNF‐alpha reduces adiponectin secretion, a hormone that improves insulin sensitivity, further contributing to insulin resistance [55].

IL‐6, produced by various cells including adipose tissue, is also linked to obesity and insulin resistance. IL‐6 triggers insulin resistance by disrupting insulin signaling and glucose transport, primarily through downregulation of IRS‐1 and GLUT‐4. IL‐6's role in insulin resistance is exacerbated by its interaction with other cytokines like TNF‐alpha and IL‐8, which are elevated in adipocytes of insulin‐resistant individuals [56].

### 6.4 Oxidative Stress

Elevated oxidative stress levels are consistently associated with increased morphometric measures such as BMI and waist‐hip ratio [53, 56]. F2‐isoprostanes, markers of lipid oxidation, are utilized to evaluate oxidative stress and may predict obesity‐related cardiovascular issues. Despite their potential, their specificity and sensitivity need further validation. Glutathione peroxidase offers strong anti‐inflammatory and anti‐atherosclerotic benefits. Additionally, complement factor 3 and monocyte chemoattractant protein‐1 (MCP‐1) are linked to excessive fat accumulation, atherosclerosis, and heightened cardiovascular risk [56].

F2‐isoprostanes, key byproducts of oxidative stress, are elevated in obese individuals, including children and those with severe obesity, compared to nonobese individuals. This elevation suggests increased oxidative damage in obesity. Measurement of F2‐isoprostanes, such as 8‐oxo‐dG, serves as a reliable marker for DNA oxidation and obesity‐related complications. Obesity‐induced oxidative stress and inflammation further enhance F2‐isoprostane production, contributing to insulin resistance and cardiovascular risk factors [57].

### 6.5 Blood Cell Profile

Among individuals with morbid obesity, there were significantly raised platelet counts, plateletcrit (PCT), and platelet‐to‐lymphocyte ratio (PLR) readings. Additionally, the obese group exhibited larger and statistically significant white blood cell counts and red cell distribution width (RDW) values [58]. Plasma PCT may serve as an indicator of inflammation. Since adipose tissues produce calcitonin mRNA, they have been regarded as endocrine organs. Additionally, it was shown that activated macrophages caused adipocytes to excrete procalcitonin in vitro, and it has been suggested that the number of these macrophages in body fat is proportionally associated with the magnitude of obesity. Procalcitonin has a strong positive correlation with BMI z‐score [59]. Studies have shown that obesity is linked to an upsurge in the production of inflammatory mediators within adipose tissue, resulting in inflammation. The degree of this inflammatory response appears to be directly correlated with the quantity of adipose tissue [60].

## 7 Omics Biomarker: Use of Omics Technologies in Obesity Research

**FIGURE 3:**
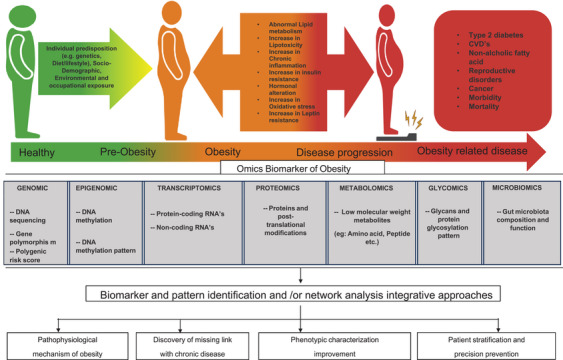
Exploring multi‐omics biomarkers in obesity research. Insights from genomics, proteomics, and metabolomics. This figure illustrates the progression from a healthy state to obesity‐related diseases, highlighting key metabolic changes. It categorizes obesity biomarkers into genomic, epigenomic, transcriptomic, proteomic, metabolomic, glycomic, and microbiomic levels, emphasizing their role in biomarker identification, disease characterization, and precision prevention strategies.

The identification of novel “omics” biomarkers holds promise for advancing our comprehension of obesity's pathophysiology and its connections to chronic diseases as shown in Figure 3 [60]. Moreover, the utilization of “omics” biomarkers could enhance the precise delineation of obesity profiles and suggest potential areas for customized prevention and treatment strategies [61]. The investigation of genes (genomics), messenger RNA (mRNA) and miRNAs (transcriptomics), proteins (proteomics), and metabolites (metabolomics) has accrued significant attention in recent times [62]. Additional “omics” technologies that provide perspectives on modulation of biological pathways encompass gut microbiota and epigenomics, which utilize DNA methylation as a marker for gene expression and phenotype (microbiomics). Numerous analytical methodologies and bioinformatic utilities have been created to analyze the vast amount of data generated from these approaches, including omics‐based techniques, pathway analyses, and network studies. More recently, integrative approaches like multi‐omics and trans‐omics studies have emerged as prominent trends in the field [63]. Table 2 highlights the various omic biomarkers and their mechanisms of action, providing a comprehensive overview of their roles in obesity research and management.

**TABLE 2:** List of various biomarkers related to obesity their association and mechanism.

Sr no.	Biomarker type	Specific biomarker	Association	Mechanism	References
1.	MicroRNA	miR‐222, miR‐142‐3, miR‐140‐5p, and miR‐143	Obesity, NAFLD, and/or insulin resistance in children. Prospective cardiovascular disease and other illnesses' diagnostic indicators.	Extracellular glucose‐induced upregulation in 3T3‐L1 (3T3‐L1 preadipocyte cell line) adipocyte cells increases insulin resistance	[41, 64]
		PTEN gene (hsa‐miR‐130b‐3p, hsa‐miR‐142‐5p, hsa‐miR‐148a‐3p, hsa‐miR‐21‐5p, hsa‐miR23a‐3p, hsa‐miR‐26b‐5p, hsa‐miR‐320a and hsa‐miR‐ 486‐5p)		PTEN loss in spontaneous mutations impacts the PI3K/AKT/mTOR pathway as it is situated downstream of the insulin pathway. The absence of PTEN disrupts the regulation of this pathway, leading to unrestricted signal flow even when serum insulin levels are low.	[49, 50]
2.	Adipocytokines	Plasminogen Activator Inhibitor‐1 (PAI‐1)	Cardiovascular disorders, metabolic issues associated with obesity	PAI‐1 is responsible for the natural inhibition of the fibrin breakdown process. It achieves this by inhibiting the activity of tissue plasminogen activator and urokinase‐type activator, leading to the accumulation of fibrin in the basement membranes and interstitial tissues.	[56, 65]
3.	Oxidative stress	F2 isoprostanes	Cardiovascular diseases brought on by obesity	F2‐isoprostanes, known indicators of oxidative stress, tend to be more concentrated in the blood following a sudden rise in plasma lipids, particularly among individuals with obesity and hypertension.	[66]
		Glutathione peroxidase	Obesity lead Atherosclerosis and CVD	In reaction to the heightened oxidative stress induced by homocysteine on endothelial function and atherosclerotic lesion, GPx‐1 acts as a protective signal. In individual suffering from coronary artery disease the erythrocyte GPx‐1 base line level is inversely linked to future cardiovascular risk (CAD).	[67]
		Monocyte chemoattractant protein‐1 (MCP‐1)	Obesity and elevated risk of cardiovascular disease	MCPIP (MCP‐1‐induced protein), a zinc finger protein, stimulates 3T3‐L1 cells adipogenesis without relying on PPARgamma activation.	[59, 68]
4.	Blood cell profile	Plateletcrit (PCT)	Increased thrombotic state and inflammatory response in patients with morbid obesity.	Plasma procalcitonin (PCT) levels are indicative of inflammation, with adipose tissue acting as an endocrine organ that produces calcitonin mRNA. In vitro studies show that adipocytes release procalcitonin in response to activated macrophages. A positive correlation exists between BMI z‐score and procalcitonin levels, establishing a direct relationship between increased adipose tissue volume and heightened production of inflammatory mediators in obesity‐induced inflammation.	[59, 64]
5.	Genomics	Leptin	Sympathetic nervous system (SNS) activity	Adipose tissue is responsible for releasing leptin, which influences the heightened activity of the sympathetic nervous system (SNS) leading to renin–angiotensin–aldosterone system (RAAS) activation. The visceral fat and increased renal sinus fat around the kidney activates RAAS causing physical compression of the kidneys.	[69]
		Melanocortin‐4‐receptor gene (MC4R)	Energy homeostasis, food intake, and body weight regulation at the hypothalamus level	MC4R controls the leptin‐pro‐opiomelanocortin pathway, which regulates both food intake and energy expenditure, resulting in elevated plasma insulin levels and reduced insulin tolerance	[70]
6.	Epigenomics	DNA methylation	Identification of high‐risk individuals, obesity subtypes, and treatment monitoring	DNA methylation can reveal genomic changes linked to inflammation, but the exact mechanisms and causal relationships need further exploration. Several methylation sites associated with obesity have been identified, though some findings require replication.	[71]
		Histone modifications	Epigenetic regulation of adipogenesis	High‐fat diets and fasting affect histone deacetylases (HDACs), which regulate gene expression. In the medial hypothalamus, histone acetylation alters neuropeptide expression controlling feeding, metabolism, and reproduction. During adipocyte differentiation, histone modifications regulate key adipogenesis genes, including Pref‐1 (Pre‐adipocyte factor 1), C/EBP (CCAAT/enhancer‐binding protein), PPAR, and aP2 (adipocyte fatty acid‐binding protein).	[72]
7.	Transcriptomes	Circulating miRNAs	Obesity and obesity‐associated comorbidities	In children and adolescents with obesity, miR‐222, miR‐142‐3, miR‐140‐5p, and miR‐143 are upregulated, affecting hepatic function and cholesterol synthesis. Elevated miR‐122 levels are linked to liver steatosis, NAFLD progression, and increased ALT, AST, GGT, triglycerides, and reduced HDL cholesterol.	[41]
		Long ncRNAs (lncRNAs)	BMI	A functional long noncoding RNA (lncRNA) derived from the CEBP locus plays a role in adipogenesis by preventing methylation of the CEBP gene, resulting in increased CEBP mRNA expression	[73]
8.	Proteomics	S100‐A8 and S100‐A9	BMI	S100A8 and S100A9 are highly expressed in inflamed tissue fluid, playing a key role in conditions like psoriatic and rheumatoid arthritis. Obesity, linked to elevated psoriasin levels, increases the risk of developing psoriasis in individuals with a BMI over 30.	[74]
9.	Metabolomics	Acylcarnitines	Insulin resistance in obesity	Changes in carnitine (CN) levels indicate disruptions in mitochondrial β‐oxidation, leading to the buildup of free fatty acids and acyl‐CoA. This backlog results in accumulated short‐chain carnitines and contributes to insulin resistance.	[75]
		Branched‐chain amino acids (BCAA)	Oxidative stress related to obesity	Elevated branched‐chain amino acids (BCAAs) contribute to obesity and related diseases by promoting apoptosis, oxidative stress, mitochondrial dysfunction, and activating the mTOR signaling pathway.	[76]
10.	Lipidomics	Lysophosphatidylcholine	Negative correlation between BMI and waist circumference and a positive correlation with high‐density lipoprotein cholesterol	Obesity reduces lysophosphatidylcholine levels and disrupts lysophospholipid metabolism, impairing response to n–3 PUFAs. In HepG2 cells, noncytotoxic steatosis alters lysophospholipid secretion, reflecting changes seen in obese individuals' plasma.	[77]
11.	Glycomics	IgG N‐glycosylation pattern	Central adiposity and obesity	The reduction of galactosylation due to the proinflammatory activity of IgG.	[78]
12.	Microbiomics	Firmicutes/Bacteroidetes ratio	Change in gut microbiota of obesity patient	Obesity leads to an increase in Firmicutes and a decrease in Bacteroidetes, with reduced bacterial diversity compared to lean individuals. These changes suggest more significant alterations at the family, genus, or species level beyond the Firmicutes/Bacteroidetes ratio.	[79]
		N‐glycosylation by oligosaccharyltransferase	Mouse apoptosis inhibitor of macrophage (AIM)	Obesity reduces N‐glycosylation by oligosaccharyltransferase, affecting scavenger receptor AIM. Removal of N‐glycans decreases AIM secretion and promotes its integration into adipocytes, increasing AIM lipolytic activity.	[80]

### 7.1 Genomics

Hereditary factors contribute 40%–70% to obesity risk, as extensively studied through twin and adoption research since the 1970s [81]. The discovery of leptin and its receptor genes, along with the leptin‐driven melanocortin 4 signaling pathway, led to investigations into genetic causes of early‐onset severe obesity. These rare monogenic forms often involve disruptions in the leptin‐melanocortin pathway, crucial for appetite regulation and energy homeostasis [82, 83]. Leptin's role extends to increasing sympathetic nervous system activity through the renin–angiotensin–aldosterone system, impacted by visceral fat surrounding the kidneys [69]. Most obesity cases are multifactorial, influenced by multiple genetic variations [83, 84]. Recent advancements in genomic research, particularly DNA microarrays and NGS, have enabled detailed mapping and exploration of population‐specific genetic characteristics [84]. Genome‐wide association studies (GWAS) have identified numerous genetic variants associated with obesity, involving pathways related to food intake, energy expenditure, adipocyte maturation, and insulin signaling [85, 86, 87]. A recent GWAS with 700,000 participants identified 941 SNPs linked to BMI, with key genes like FTO and MC4R contributing to polygenic obesity [88, 89, 90]. However, these loci collectively explain less than 3% of BMI variability, partly due to the challenges in detecting significant loci [88]. Genetic risk scores (GRS) have been developed to predict obesity risk using composite genetic profiles. A new GRS, incorporating 2.1 million genetic variants, provides more accurate predictions of BMI compared to previous models [91]. This GRS, with a correlation coefficient of 0.29, outperforms earlier models and could enhance personalized obesity prevention strategies and lifestyle interventions, especially for individuals with higher genetic risk [91, 92].

### 7.2 Epigenomics

Recent advancements in epigenomics have enhanced our understanding of how epigenetic regulation, including DNA methylation, histone modifications, and noncoding RNAs (ncRNAs; e.g., miRNAs), influences obesity and related conditions [93]. DNA methylation at CpG dinucleotides, modifiable by environmental factors, has been linked to obesity, although more replication studies are necessary to confirm these findings [71, 94]. Epigenetic biomarkers, affected by early‐life stress and nutrition, can predict obesity risk in later life. For instance, the Dutch Hunger Winter study demonstrated significant DNA methylation changes due to prenatal famine exposure [95, 96]. Epigenome‐wide association studies (EWAS) have identified correlations between DNA methylation patterns and obesity traits, but the high rate of false positives underscores the need for further validation [97, 98]. Research on histone modifications in obesity is limited due to methodological constraints, though existing studies suggest connections between histone modifications and metabolic profiles [99]. Advanced techniques like ChIP sequencing are needed to explore histone modifications and their role in obesity further. Establishing causality between epigenetic changes and obesity remains challenging due to the dynamic nature of epigenetic modifications [100].

### 7.3 Transcriptomics

Transcriptomics bridges the gap between GWAS and physiological research by decoding gene‐related information. RNA sequencing and array‐based methods identify both protein‐coding mRNAs and ncRNAs [101, 102]. In obesity research, analyses of adipocytes from visceral and subcutaneous fat reveal altered expression in over a thousand genes compared to lean individuals [103]. Array‐based gene expression profiling of whole‐blood mRNA levels from two independent cohorts (KORA F4 and SHIP‐TREND) identified associations with BMI. These gene expression patterns were linked to crucial metabolic pathways, including protein synthesis, cell death due to inflammation or lipotoxicity, insulin signaling, and oxidative stress [103]. ncRNAs, including miRNAs and long ncRNAs (lncRNAs), play essential roles in post‐transcriptional regulation. Differentially expressed miRNAs in adipose tissue contribute to adipogenesis, differentiation, and insulin signaling. cmiRNAs in blood are promising biomarkers due to less invasive collection methods and their role in metabolic crosstalk. A study found 33 dysregulated cmiRNAs associated with PI3K‐Akt and fatty acid metabolism in obese individuals [49]. LncRNAs also influence adipogenesis, inflammation, and insulin sensitivity [104, 105]. Challenges in transcriptomics include tissue‐specific variation and difficulties in isolating and analyzing cmiRNAs. Variations in extraction and quantification methods can affect results. Further research on ncRNAs, using both experimental and bioinformatic approaches, is needed to explore their therapeutic and preventive potential [106].

### 7.4 Proteomics

Proteomics has emerged as a key technology for identifying and characterizing proteins associated with obesity and related conditions, offering insights into protein interactions and post‐translational modifications not revealed by genomics or transcriptomics. Key bioanalytical platforms include protein microarrays, LC‐ESI‐MS, SELDI‐TOF‐MS, and MALDI‐TOF‐MS [107]. Secreted proteins, representing about 10% of the human genome, are particularly relevant in serum/plasma proteomics for understanding obesity's pathophysiology [108]. Despite challenges like small sample sizes and low repeatability in population‐based studies [109, 110], significant progress has been made. For instance, a study involving 1002 obese and overweight individuals identified several proteins, including CRP, proline‐rich acidic protein 1 (PRAP1), and complement factors B, H, and I, as well as the S100‐A8 and S100‐A9 proteins, with notable associations to BMI [111]. CRP, in particular, showed the strongest correlation with BMI, highlighting its potential role in chronic inflammation related to obesity. Further large‐scale proteomic studies are needed to identify distinct proteins that could serve as biomarkers for obesity and its associated conditions [111].

### 7.5 Metabolomics

Metabolomics involves comprehensive analysis of all metabolites in a biological system, including lipids, amino acids, peptides, organic acids, and carbohydrates [112]. It utilizes untargeted and targeted methodologies with technologies like nuclear magnetic resonance (NMR) spectroscopy and mass spectrometry (MS). Untargeted metabolomics explores thousands of metabolites simultaneously, while targeted metabolomics quantifies a selected set of metabolites. Research using both approaches has identified metabolic differences between obese and lean individuals. Notable findings include elevated plasma levels of branched‐chain amino acids (BCAA), aromatic amino acids, and acylcarnitines, as well as reduced glycine concentrations in obesity [113, 114]. Elevated BCAA levels are associated with increased risk of type 2 diabetes and insulin resistance, potentially through mechanisms involving apoptosis, oxidative stress, mitochondrial dysfunction, and mTOR signaling pathway activation [76]. Metabolomics offers significant potential for advancing precision medicine in metabolic diseases such as obesity by improving patient categorization and monitoring. It also provides valuable insights for drug discovery and development [115].

### 7.6 Lipidomics

Lipidomics, a subset of metabolomics, focuses on analyzing lipid species across various classes, including fatty acyls, glycerolipids, glycerophospholipids, sterols, and prenols [116]. Understanding the dynamic changes in these lipids in response to physiological and environmental factors is complex and requires advanced bioanalytical techniques for both untargeted and targeted analyses [117]. Traditional lipid profile screening has been used to detect dyslipidemia in obesity and related chronic diseases [118]. However, advancements in MS have identified additional plasma lipids that mediate metabolic dysfunction in obesity. For instance, elevated levels of short‐ and medium‐chain acylcarnitines in obese individuals are linked to disruptions in fatty acid synthesis and oxidation [119]. Increased plasma concentrations of free fatty acids, particularly proinflammatory omega‐6 fatty acids, are also observed in obesity [120].Targeted metabolomics studies, such as the EPIC‐Potsdam study, have shown specific phospholipid associations with obesity. Diacyl‐phosphatidylcholine levels correlate positively with BMI and waist circumference, whereas acyl‐alkyl‐phosphatidylcholines and lysophosphatidylcholines are negatively correlated [114]. These studies highlight the complex relationship between lipid profiles and obesity‐related dyslipidemia [121]. Additionally, high‐fiber diets have been found to impact phospholipid metabolic profiles in obese individuals [122]. Lipidomics continues to provide valuable insights into the role of lipids in obesity, offering opportunities for targeted interventions and therapeutic research.

### 7.7 Glycomics

Approximately 70% of human proteins are glycosylated, with the primary types being N‐linked and O‐linked glycans [123]. The glycosylation process, involving about 700 proteins, is complex and less straightforward than protein synthesis [124]. This post‐translational modification plays crucial roles in biological functions, and changes in glycosylation patterns during disease progression have become a significant focus in glycome research [124, 125]. However, glycomic databases are still developing due to the intricate and diverse nature of glycans compared to other “‐omics” fields [126].

Techniques like Capillary Electrophoresis (CE)‐ESI‐MS and LC‐ESI‐MS are commonly used in glycomic studies to analyze glycan structures [123]. Research has shown that alterations in glycosylation patterns, such as reduced galactosylation of IgG, are associated with central adiposity and obesity [78, 127, 128]. Reduced galactosylation is linked to increased proinflammatory activity of IgG, which may contribute to the chronic inflammation seen in obesity. Thus, glycomics research has the potential to uncover novel inflammatory biomarkers involved in the onset and progression of obesity.

### 7.8 Microbiomics

The human microbiome comprises the total bacterial population associated with the human body [129]. The term “dysbiosis” describes an imbalance in the microbiota's composition and function [130, 131]. Microbiomic studies use techniques like 16S rRNA gene sequencing and metagenomics to explore microbial structure, function, and composition, while microarray‐based technologies aid in meta‐transcriptomic analysis [132]. Recent research employs metabolomic and multi‐omics approaches to assess the microbiome's role and interactions with the host [132, 133].

Obesity is often linked with altered gut microbiota, notably an increased Firmicutes/Bacteroidetes ratio [134]. However, the relationship between obesity and microbiota composition is complex and not fully conclusive [135, 136]. Metagenomic studies reveal that the functional diversity of the gut microbiota plays a crucial role in obesity, emphasizing that microbiome function is as important as its composition [80].

Short‐chain fatty acids (SCFAs) such as acetate, propionate, valerate, and butyrate, produced from dietary fiber fermentation, are typically elevated in obese individuals and may influence metabolism, appetite, and immune response [137, 138]. Dysbiosis induced by early‐life antibiotics has been linked to increased obesity risk in children and adolescents through effects on metabolic species, calorie absorption, hepatic function, hormone secretion, and metabolic signaling [139]. Probiotics, synbiotics, and prebiotics show potential in managing obesity, offering benefits like weight loss and reduced inflammation [140]. Helicobacter pylori, affecting gastrointestinal hormones like leptin and ghrelin, may influence food intake and energy balance, though its precise role remains under investigation [141]. Table 3 summarizes the mechanisms of action of various biomarkers studied in obesity through preclinical animal trials and clinical investigations. These biomarkers play pivotal roles in elucidating the pathophysiology of obesity, including metabolic dysregulation, inflammation, and hormonal imbalances. For example, adipokines such as leptin and adiponectin regulate energy homeostasis and lipid metabolism, while inflammatory markers like TNF‐α and IL‐6 highlight the role of chronic inflammation in obesity. Gut microbiota–derived metabolites, including SCFAs, provide insights into microbial contributions to obesity. These studies emphasize the translational potential of these biomarkers in developing targeted therapies and personalized interventions for obesity management. Future microbiomic research is likely to focus on functional aspects of the microbiome, potentially unveiling new strategies for obesity prevention and treatment.

**TABLE 3:** Mechanism of action of different biomarkers used in obesity as studied in preclinical animal trials/ clinical trial.

Study	Trail design	Intervention	Outcome	Mechanism of action	Relevance	Reference
**MicroRNA biomarker**						
Liver microRNA transcriptome reveals miR‐182 as link between type 2 diabetes and fatty liver disease in obesity	**Human study**: Case‐control, comparing 20 obese individuals with T2D and 20 without, using liver biopsies for microRNA microarray analysis. **Animal study**: Diet‐induced obese mice with hepatic overexpression of miR‐182–5p, followed by dietary interventions and weight loss treatments.	MicroRNA microarray analysis, validation in human liver samples	miR‐182‐5p links T2D and fatty liver disease in obesity	LRP6 (Low‐density lipoprotein receptor‐related protein 6) repression by miR‐182‐5p impairs glucose homeostasis, promotes lipogenesis	Potential as a biomarker and therapeutic target for obesity‐driven hepatic steatosis	[142]
Plasma Exosomes in Obesity‐driven Diabetes Exacerbate Progression of Triple Negative Breast Cancer	**Animal study**: Diet‐induced obesity mouse model to study plasma exosome effects. **Cell study**: In vitro TNBC cell culture exposed to plasma exosomes from obese mice.	High‐fat diet (HFD)	HFD‐derived exosomes increase metastatic burden in lung/brain	HFD‐derived exosomes reprogram gene expression, promoting EMT (Epithelial‐Mesenchymal Transition) in TNBC cells (Triple‐Negative Breast Cancer cell)	Potential microRNA biomarkers for obesity‐related cancer progression	[143]
Upregulated miR‐205 as a biomarker of diet‐induced obesity‐related glomerulopathy	Randomized controlled trial with 30 Wistar rats, divided into two groups: 15 rats on a standard diet (SD) and 15 rats on a high‐fat diet (HFD) for 10 weeks.	High‐fat diet (HFD) vs. Standard diet (SD)	miR‐205 upregulated in urine, potential biomarker for early ORG	HFD rats developed early‐stage ORG with miR‐205 upregulation	miR‐205 in urine as a biomarker for obesity‐related glomerulopathy	[144]
Differential microRNA expression profile in obesity‐induced kidney disease driven by high‐fat diet in mice	Male C57BL/6J mice, divided into two groups: standard diet (STD) and high‐fat diet (HFD) for 10 weeks.	High‐fat diet (HFD)	Evaluation of kidney histopathology (PAS and Oil‐Red‐O staining), gene expression of inflammatory, profibrotic, and lipid metabolism markers via real‐time PCR, and miRNA differential expression analysis using miRNA sequencing. Bioinformatic analysis (KEGG/GO) was performed to identify target genes involved in metabolic and inflammatory pathways.	Differentially expressed miRNAs play roles in obesity‐associated kidney disease	miRNAs as potential biomarkers for obesity‐related kidney dysfunction	[145]
microRNA Expression Profile in Obesity‐Induced Kidney Disease Driven by High‐Fat Diet in Mice	Male C57BL/6J mice were fed either a standard or high‐fat diet for 10 weeks to assess kidney histopathology, gene expression, and miRNA profiles, identifying potential therapeutic targets for obesity‐related kidney disease.	High‐fat diet (HFD)	miR‐5099, miR‐551b‐3p, miR‐146a‐3p significant in expression differences	Validated miRNAs involved in obesity‐induced kidney disease pathways	miR‐5099, miR‐551b‐3p, miR‐146a‐3p as potential biomarkers in preclinical obesity research	[146]
Tissue and Circulating MicroRNAs 378 and 142 as Biomarkers of Obesity and Its Treatment Response	Study assessed the expression of miR‐378 and miR‐142 in subcutaneous adipose tissue (SAT) and plasma of 51 obese patients and 10 healthy controls, before and during sibutramine therapy, to explore the molecular effects of obesity duration and treatment on adipogenesis and fibrogenesis.	Real‐time PCR assessment of miR expression	miR‐378 increased in early obesity, miR‐142 decreased with duration of obesity	Changes in miR‐378 and miR‐142 levels reflect obesity stage and treatment response	miR‐378 and miR‐142 as biomarkers for obesity and its treatment response	[147]
**Inflammatory biomarkers**						
The Potential Link between Obesity, Synbiotics Intake and Inflammasomes in an Animal Model	Study involved 30 male Wistar rats divided into four groups: normal control, HFD, normal + synbiotics, and HFD + synbiotics. After 8 weeks, rats were sacrificed, and tissue samples were collected to assess body weight changes and NLRP3 expression using real‐time PCR.	High‐fat diet (HFD) with/without synbiotics	Synbiotics reduced NLRP3 (NLR family pyrin domain containing 3) expression in adipose and liver tissues	Synbiotics disrupted inflammatory consequences of obesity by targeting NLRP3	Potential therapeutic benefits in reducing obesity‐related inflammation	[148]
Visualization of macrophage inflammatory activity on visceral obesity in high‐fat diet‐induced obese mice	Male C57BL/6 mice were fed a high‐fat diet (60% fat) for 20 weeks to induce obesity. Insulin tolerance, CRP levels, and macrophage inflammatory activity (measured by SUVmax on 18F‐FDG PET/CT) were assessed. Flow cytometry, histology, and molecular analyses were performed on harvested visceral adipose tissue (VAT) after sacrifice.	18F‐FDG PET/CT (Fluorine‐18 Fluorodeoxyglucose Positron Emission Tomography/Computed Tomography) imaging for VAT inflammation assessment	VAT SUVmax increased, correlated with CRP levels and macrophage infiltration	Obesity‐induced VAT inflammation visualized by 18F‐FDG PET/CT	Potential for assessing inflammatory biomarkers in obesity	[149]
Systemic inflammation markers of diet‐induced metabolic syndrome in rat model	Thirty‐three male Wistar rats were divided into control (*n* = 15) and experimental (MS) groups (*n* = 18), with the MS group receiving a high‐fat/high‐carbohydrate diet for 12 weeks. Systemic inflammation and metabolic impairments were assessed by measuring white blood cell counts, serum levels of total protein, C‐reactive protein, cytokines (IL6, IL10, TNFα), insulin, leptin, and reactive oxygen species production in adipose tissue.	Diet‐induced metabolic syndrome, measured systemic inflammation	Systemic inflammation markers increased, including CRP, IL10, and TNFα	High‐fat/high‐carb diet induced systemic inflammation	Relevance of systemic inflammation markers in preclinical assessment of obesity‐related metabolic syndrome	[150]
**Adipocytokines biomarkers**						
Importance of adipocytokines in obesity‐related diseases	Male mice were fed a high‐fat, high‐sucrose diet, and biological outcomes, including glucose metabolism, insulin resistance, and vascular changes (intimal thickening), were assessed	Gene expression profile analysis of visceral and subcutaneous fat	Adiponectin plays a key role in metabolic syndrome and atherosclerosis	Hypoadiponectinemia linked to CAD and insulin resistance	Understanding the role of adipocytokines in obesity‐related conditions	[151]
**Oxidative stress biomarker**						
Study of oxidative stress biomarkers in obese children	The study investigated oxidative stress, inflammation, and dysregulation of adipocytokines in adults and children with obesity. The trial measured reactive oxygen and nitrogen species, antioxidant enzyme activity, and adipocyte dysfunction to establish their association with obesity‐related metabolic disturbances	N/A	Higher MDA levels and lower Vitamin C levels in obese children	Oxidative stress measured by increased MDA and decreased Vitamin C levels	Importance of oxidative stress biomarkers in pediatric obesity	[152]
Role of Oxidative Stress on Insulin Resistance in Diet‐Induced Obesity Mice	Diet‐induced obese mice (insulin‐resistant) were treated with N‐acetylcysteine (NAC, 50 mg/kg/day for 15 days). The effects on insulin resistance, fasting glycemia, oxidative stress (measured by DCF oxidation, CAT activity, and GSH levels), and molecular markers (NFκB, PTP1B, JNK phosphorylation, IRS, Akt, and IRS/PI3k association) were assessed post‐treatment. Biochemical and molecular analyses were conducted on tissues from euthanized mice.	N‐acetylcysteine (NAC) treatment for 15 days	Improved insulin resistance, reduced oxidative stress markers	NAC treatment reverses oxidative stress and improves insulin signalling	Potential therapeutic role of NAC in reducing insulin resistance in obesity	[153, 154]
**Genomics biomarker**						
Small Animal Models of Obesity	Literature review on the use of small rodents as animal models to study obesity induced by fat‐rich diets, with a focus on their similarities and differences to human obesity. The review examines physiological, hormonal, and behavioral mechanisms involved in fat‐induced obesity, including nutrient utilization efficiency, hormonal roles (leptin, ghrelin, insulin), and eating behaviors. The chapter also discusses the potential for reversing obesity and provides recommendations for future research and study design in animal models of obesity.	Genomic biomarkers, high‐throughput gene expression analysis	Enhanced understanding of obesity and metabolic function	Technological advances in genetic tools and molecular genetic tools	Impact on obesity research and body weight homeostasis	[155]
Analysis of a Genetic Region Affecting Mouse Body Weight	Inbred BTBR and C57BL/6J mice were used to identify genes affecting obesity and glucose metabolism. A ∼316 kb region on chromosome 2 was studied, focusing on Pdk1 and Itga6. Knockout mice lacking Pdk1 or Itga6 were fed an obesogenic diet to assess their role in obesity and glucose tolerance.	Knockout mice (Pdk1, Itga6)	Identified genetic region influencing obesity and lipid metabolism	Pdk1 role in cardiac lipid metabolism	Insights into genetic regulation of obesity	[156]
Early Hepatic Proteomic Signatures in High‐Fat‐Induced Obesity	Rats were fed either a normal diet or a high‐fat diet (HFD) for 24 weeks to study early hepatic changes in obesity. Hepatic protein profiles were analyzed using two‐dimensional differential gel electrophoresis (DIGE) and identified by MALDI‐TOF MS.	High‐fat diet	Differentially expressed proteins (18 proteins, 2‐ to 4‐fold change) involved in lipid metabolism, energy metabolism, detoxification, urea cycle, and hepatic Ca homoeostasis were identified.	Western blot and immunohistochemistry showed increased expression of liver‐specific arginase‐1 (Arg‐1) in HFD rats, correlating with NASH‐related fibrosis in humans.	Potential therapeutic targets for obesity‐related liver disease	[157]
Integrative Analysis of Multi‐Omics Data	A multi‐omics atlas was created for wheat, including 132,570 transcripts, 44,473 proteins, 19,970 phosphoproteins, and 12,427 acetylproteins. The analysis identified key transcriptional networks, PTM impacts, and genes involved in development and disease resistance, highlighting TaHDA9‐TaP5CS1 as a regulator of Fusarium crown rot resistance via proline accumulation.	Multi‐omics data integration	Identified DEHGs, DMRs, DAMs for obesity	Mendelian Randomization and causal pathway analysis	Insights into genetic factors contributing to obesity	[158]
Genetically Engineered Mice in Obesity Research	Transgenic mice overexpressing glycerol 3‐phosphate dehydrogenase and carrying ALBP promoter/enhancer‐reporter constructs were generated to study obesity mechanisms. Additionally, transgenic and gene knockout mice were used to identify mutations causing single‐gene obesity syndromes and for pharmaceutical drug development targeting human‐specific drug pathways.	Gene targeting, transgenics	Identified gene mutations causing obesity syndromes	Dysregulation of homeostatic mechanisms	Understanding single gene obesity syndromes	[159]
Transcriptome‐Wide Analyses of Adipose Tissue	Outbred HS rats (415 males) were used for RNAseq analysis of adipose tissue to identify adiposity‐associated genes and gene networks. Weighted gene coexpression network analysis (WGCNA) and mediation analysis were employed to correlate gene expression with body weight (BW) and to identify genes driving adiposity. The rat data was compared to human cohorts to identify consensus genes associated with BMI and BW, highlighting genes involved in lipid metabolism, inflammation, and adipogenesis.	RNAseq data analysis	Identified 554 consensus genes correlating with body weight	Gene co‐expression network and mediation analysis	Understanding genetic regulatory mechanisms relevant for human obesity	[160]
**Transcriptomics biomarker**						
Integrated Transcriptomic Landscape of Anti‐Steatotic Treatments	Systematic review and meta‐analysis of transcriptomic data from GEO database comparing hepatic gene expression in wild‐type HFD‐fed C57BL/6J mice versus control (ND) mice, and HFD‐fed mice treated with anti‐steatotic agents. Differential gene expression analysis was performed to identify the hepatic gene expression signature of HFD mice and assess the modulation of this signature by anti‐steatotic treatments.	Anti‐steatotic treatments	Identified 62 common genes between HFD models and human NAFLD, with 10 genes reversed by treatments	Transcriptomic biomarkers indicating potential for obesity treatment	Highlights commonalities between HFD mouse models and human NAFLD	[161]
Full Transcriptome Analysis in Obesity Models	A 46‐day experiment involving 32 male C57BL/6J mice and 24 male genetically obese db/db mice, fed a high‐fat and fructose diet. Mice were treated with quercetin (Q) at doses of 25 or 100 mg/kg. The study analyzed differential expression of 39,430 genes in mouse livers using microarray‐based transcriptome profiling (Agilent One‐Color Microarray). Bioinformatics analysis in the “R” environment was used to identify metabolic pathways (KEGGs) affected by Q exposure.	Quercetin treatment	Differential gene expression in liver tissue related to lipid, carbohydrate metabolism, and immune responses	Genes like Saa3, Cidec, G6pdx, Pdk4, CD14i, Jchain affected by quercetin	Insights into metabolic pathways affected by quercetin in obesity models	[162]
Distinct Regulation of Mu Opioid and Cannabinoid Receptor Genes in Obesity	Study examined transcriptional regulation and epigenetic modifications of Cnr1 and Oprm1 in hypothalamus of diet‐induced obesity rats (5 and 21 weeks on high‐fat diet) and humans with obesity. Gene expression and DNA methylation at gene promoters were analyzed to identify time‐dependent changes and potential biomarkers for obesity development.	Hypothalamic gene expression analysis	Up‐regulation of Cnr1 and Oprm1 genes in hypothalamus	Epigenetic modulation of gene promoters as obesity biomarkers	Highlights the role of Cnr1 and Oprm1 in obesity‐related signaling pathways	[163, 164]
Differentially Expressed Genes in Adipose Tissues of Rats	Male Sprague Dawley rats were fed an ordinary diet (control) or a diet with double the ordinary intake (experimental) for 2 months. Subcutaneous and visceral adipose tissues were collected for RNA extraction, and RT2 Profiler PCR Arrays were used to identify differentially expressed genes (DEGs) associated with adipose tissue accumulation.	Gene expression analysis in adipose tissue	Downregulation of BRS3 and UCP1 in visceral adipose tissues	Association with fat cell differentiation and triglyceride regulation	Identifies potential targets for obesity treatment through adipose tissue modulation	[165]

## 8 Multi‐Omics Integration in Obesity Biomarker Discovery

**FIGURE 4:**
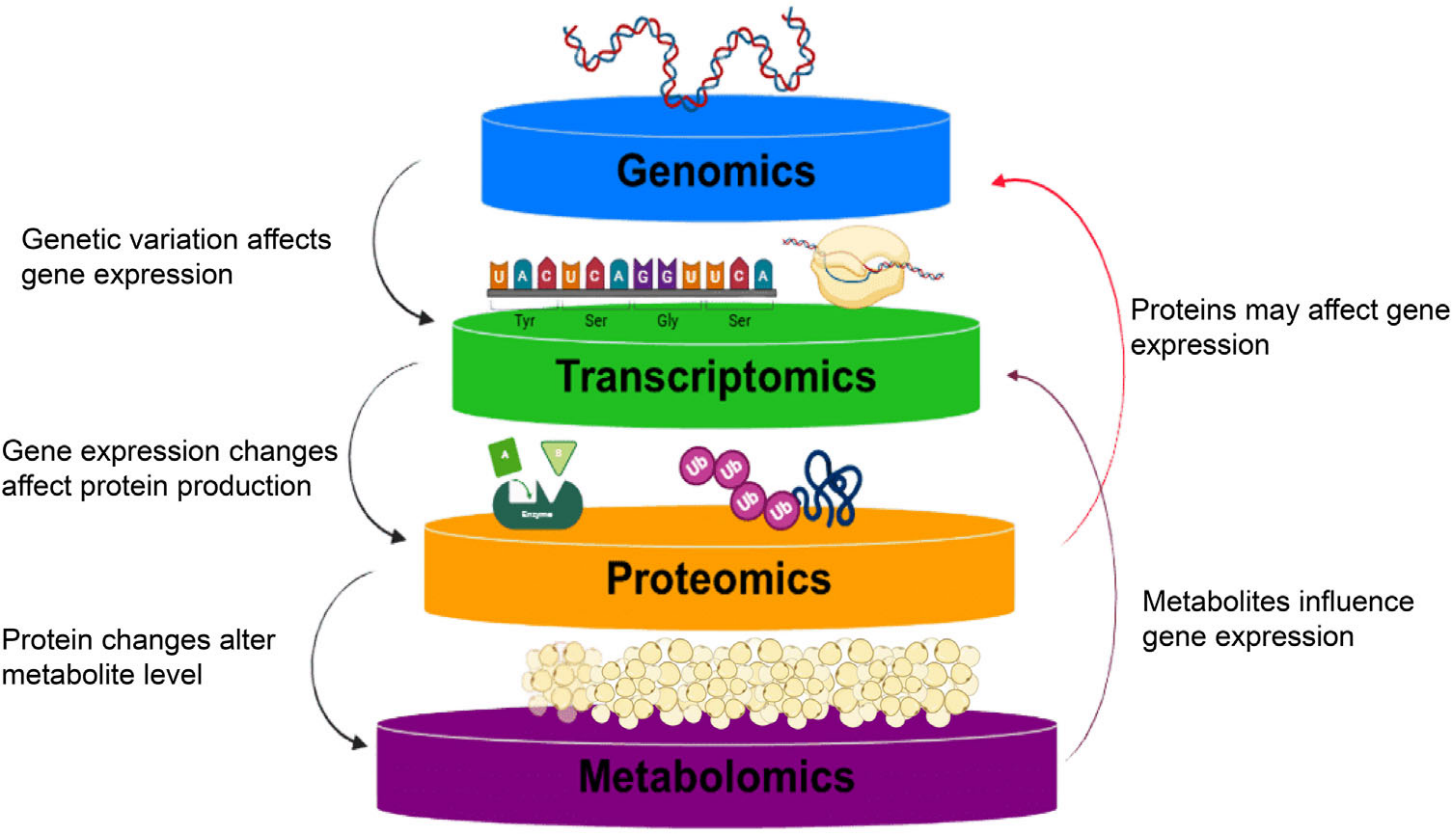
Multi‐omics interrelationships in obesity research. Schematic illustrating the interrelationships between genomics, transcriptomics, proteomics, and metabolomics in obesity research. It shows how genetic variations influence gene expression, which impacts protein production and ultimately alters metabolite levels in obesity. These integrated omics data provide insights into the molecular mechanisms underlying obesity and its biomarker.

The integration of multiple omics technologies, including genomics, transcriptomics, metabolomics, and proteomics, is revolutionizing the study of obesity by offering a comprehensive understanding of its underlying biological mechanisms. This multi‐omics approach enhances the identification of novel biomarkers for early diagnosis, personalized treatment, and better management of obesity and its associated complications [166] as illustrated in Figure 4.

A recent study demonstrated the power of multi‐omics integration in childhood simple obesity, combining transcriptomic, metabolomic, and microbiome data. This approach led to the identification of 599 differentially expressed genes, 71 differentially expressed metabolites, and 12 differentially abundant gut microbiota species. By applying a Random Forest model, 14 potential biomarkers were discovered, achieving an AUC of 0.912, which suggests their potential for early detection and treatment of childhood obesity. Importantly, the study highlighted the critical role of immune‐related genes in obesity, pointing to potential therapeutic targets [167].

Another study explored the integration of RNA‐seq and ATAC‐seq to examine the transcriptomic and epigenetic differences between subcutaneous and visceral adipose tissue (VAT). This approach revealed the transcription factor SREBF1 as a key regulator of adipocyte function, influencing over 100 adipocyte marker genes. Furthermore, by incorporating genetic data from biobank studies, the researchers demonstrated how abdominal obesity risk variants impact adipocyte gene expression, shedding light on new possibilities for obesity biomarker discovery [168].

In the broader landscape of obesity research, multi‐omics integration—spanning genomics, proteomics, and metabolomics—has proven instrumental in elucidating the complex molecular networks underpinning obesity and metabolic syndrome. Global and targeted metabolomics studies, for example, have identified distinct amino acid and acylcarnitine profiles associated with metabolic syndrome, offering new insights for patient risk stratification and personalized management [168]. Additionally, this approach is increasingly being used to explore ferroptosis, a regulated form of cell death, as a potential therapeutic target in obesity and related conditions such as type 2 diabetes and atherosclerosis [169].

The integration of omic data has also been crucial in understanding the molecular signatures of VAT in obesity and insulin resistance (IR). Recent studies have identified hub genes and transcription factors linked to VAT‐related inflammatory processes, which could be useful for risk stratification and therapeutic management of obesity and IR [170]. Moreover, the combination of miRNome, methylome, and proteomics data has revealed molecular signatures that differentiate between uncomplicated obesity and obesity with metabolic syndrome, providing valuable insights for personalized obesity management [171].

These studies underscore the transformative potential of multi‐omics integration in obesity research. By unraveling complex molecular profiles associated with obesity, this approach not only deepens our understanding of biological pathways involved in obesity, such as inflammation, adipogenesis, and metabolic dysfunction but also facilitates the discovery of biomarkers for early diagnosis. Ultimately, multi‐omics integration holds the promise of driving the development of personalized and targeted interventions, improving treatment outcomes, and advancing the field of obesity research.

## 9 ML and Artificial Intelligence and Biomarkers of Obesity

Recent advancements in analytical methods and artificial intelligence (AI) have revolutionized health monitoring and research. Sensor‐based devices, including smartphones, smartwatches, and wireless networks, enable continuous health monitoring [160, 161]. High‐throughput data from medical imaging (PET, SPECT, MRI), genomics, transcriptomics, proteomics, metabolomics, and lipidomics contribute to comprehensive insights into various pathophysiological conditions [172, 173]. The challenge now is to manage obesity and its comorbidities while uncovering their underlying mechanisms, where AI, particularly ML, plays a crucial role.

### 9.1 Background Knowledge

#### 9.1.1 Knowledge Discovery in Databases (KDD) and Data Mining

**FIGURE 5:**
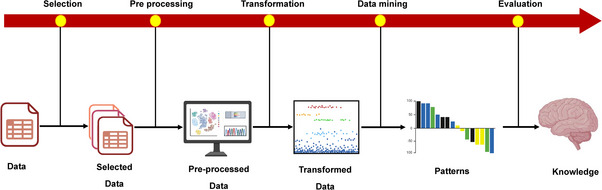
Key steps in the knowledge discovery in databases (KDD) process: From data selection to knowledge representation. The figure outlines the KDD process, detailing sequential steps including data selection, preprocessing, transformation, data mining, and evaluation, which collectively extract meaningful patterns and transform raw data into actionable knowledge.

KDD is a multistep method that focuses on data management and information extraction, as shown in Figure 5. According to Fayyad, the key phase involves data mining, which uses ML and statistical techniques to reveal these patterns [174].

#### 9.1.2 Data Selection

This initial step involves identifying and gathering relevant data from diverse sources, such as electronic health records (EHR), NGS data, and metabolomic data sets. Due to the heterogeneous nature of these data sources, they may need to be integrated into a standardized format to facilitate the KDD process. This step ensures that the data selected are pertinent to the research question and are structured in a way that supports subsequent analysis.

#### 9.1.3 Data Preprocessing

The process of preprocessing can also be denoted as data cleaning, as indicated by Fayyad et al. [174]. When data are collected from multiple sources, discrepancies in the features (descriptors) of the data can arise due to use of different units to measure the same information, such as levels of blood glucose, or omitting crucial information. This step aims to address these issues by acquiring or predicting missing data and correcting or removing inaccurate data.

#### 9.1.4 Data Transformation

To expedite the extraction process, data can be modified to produce more understandable results by employing techniques such as dimensionality reduction or variability reduction. Dimensionality reduction reduces the number of features, offering benefits such as improved data presentation, shorter analysis time, and enhanced accuracy in prediction [174]. This involves subset selection in the data set's available features that are more relevant and informative for model development. Another technique for altering data involves discretization, which simplifies the intricacy of data by transforming integer or real number values into categorical values. This is accompanied by integration of variables [175].

#### 9.1.5 Data Mining

In order to attain the intended results, an ML algorithm is employed for processing the modified data. The data mining process produces two primary categories of outcomes: informative patterns and predictive models.

#### 9.1.6 Interpretation—Evaluation

Different visualization approaches, such as graphs and plots, are utilized to present the results in the final step of the KDD process. These techniques provide the flexibility to both summarize and reach a more reliable conclusion by a subject‐matter expert like biologist and researchers.

### 9.2 ML Tools: Obesity

#### 9.2.1 Data Integration

**FIGURE 6:**
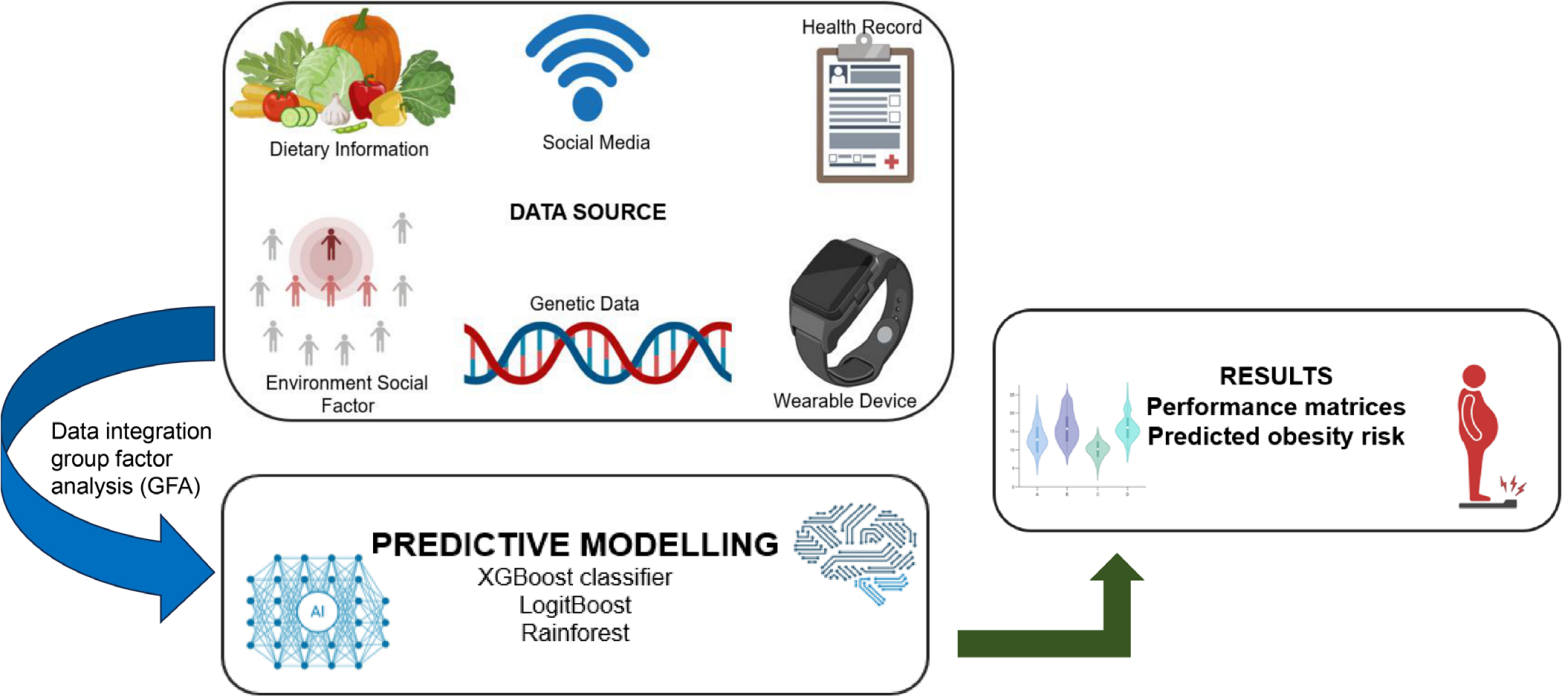
Integrative data‐driven approach for obesity risk prediction. The schematic represents a multisource data integration framework for obesity risk prediction. Various data sources, including genetic (DNA/RNA), environmental factors, wearable device metrics, dietary information, and social media data, are processed using Group Factor Analysis (GFA). Machine learning models such as Logistic Regression, XGBoost, and Random Forest are applied for predictive modeling. The results section highlights performance metrics (accuracy, sensitivity, specificity) and the predicted obesity risk, demonstrating the effectiveness of this integrative approach.

The integration of diverse data sets is a cornerstone in advancing obesity research, as it enables comprehensive insights into the multifactorial nature of the condition. For instance, combining EHR, wearable device outputs, and social media data has been instrumental in uncovering hidden patterns contributing to obesity trends, as illustrated in Figure 6 [176]. Similarly, the integration of genome‐wide SNPs, DNA methylation data, and dietary factors has facilitated the identification of complex gene–gene and gene–diet interactions, enhancing the prediction of obesity risk [177]. Group Factor Analysis (GFA) has been effectively employed to analyze multivariate data sets, such as clinical, genomic, cytokine, and dietary information, revealing molecular mechanisms associated with obesity [178]. Furthermore, incorporating individual and social/environmental factors through Community Health Surveys and geospatial data has demonstrated the influence of these dynamics on obesity prevalence [179].These integrative approaches underscore the significance of harmonizing diverse data sources to generate actionable insights, develop targeted interventions, and advance precision medicine in obesity management [180].

#### 9.2.2 Predictive Modeling

Predictive modeling using ML has emerged as a transformative approach in obesity research, leveraging advanced algorithms to enhance accuracy and interpretability. For example, hybrid models like ANN‐PSO have achieved 92% accuracy by integrating genetic, behavioral, and environmental factors to predict obesity risk effectively [181]. XGBoost classifiers have been widely utilized due to their high accuracy, as demonstrated in studies predicting obesity based on demographic and lifestyle data, achieving performance metrics of up to 96% accuracy [182]. Comparative analyses have also shown XGBoost outperforming other models like LightGBM and CatBoost in obesity risk detection with 91% accuracy [183]. ML models such as Random Forests and LogitBoost have further demonstrated the potential for forecasting obesity trends and identifying high‐risk populations, leveraging data from lifestyle habits and health records [176, 183]. Additionally, feature engineering techniques like SelectKBest have enhanced model performance, achieving classification accuracy as high as 96.68% with optimized feature subsets [184].

## 10 Challenges in ML/AI in Obesity

ML tools present significant challenges in addressing obesity, primarily due to the complexity of the disease and the variability in data quality and sources. Obesity is influenced by multifaceted interactions among genetic, environmental, and behavioral factors, making it difficult to construct generalized predictive models. While ML models, such as XGBoost and deep neural networks, have shown high accuracy in predicting obesity risk and estimating levels based on behavioral factors like diet and physical activity, their performance can be highly contingent on the data set used and the specific task at hand [185, 186]. For instance, data sets often suffer from inconsistencies, biases, and a lack of representativeness, which can skew the predictions and limit the applicability of these models across diverse populations.

Additionally, the interpretability of these models remains a significant concern. Frameworks like Explainable AI (XAI) provide tools such as SHAP analysis to enhance the understanding of model predictions and feature importance, yet the intricate nature of obesity‐related data, including nonlinear gene–environment interactions, complicates the derivation of clear, actionable insights [187]. Moreover, integrating diverse data types—ranging from genomic and epigenomic data to lifestyle and social factors—and ensuring robust model validation across different populations are ongoing challenges. These hurdles must be addressed to maximize the potential of ML in obesity management, making it imperative to prioritize the development of more adaptable, transparent, and population‐specific models [187, 188].

## 11 AI and ML in Personalized Treatment and Clinical Application

Implementing early intervention strategies involves developing personalized treatment plans based on biomarker profiles. These plans may include tailored dietary recommendations, exercise programs, or pharmacological treatments aimed at mitigating risk factors before more severe symptoms arise [189]. AI and ML are revolutionizing obesity management by integrating biomarkers to enable data‐driven, personalized interventions. Biomarkers provide insights into genetic, epigenetic, and metabolic profiles, allowing AI models to predict responses to various therapies and guide individualized treatment strategies. For instance, genetic markers can identify patients likely to benefit from specific drugs, such as appetite suppressants or metabolic enhancers, optimizing treatment efficacy and minimizing side effects. Additionally, biomarkers inform lifestyle interventions by guiding personalized diet and exercise plans [190]. ML algorithms analyze lipid profiles, inflammatory markers, and gut microbiome data to design nutrition and exercise regimens targeting metabolic pathways for effective, sustainable weight management [191].

## 12 Clinical Implications of Obesity Biomarkers

### 12.1 Early Detection and Prevention

Obesity biomarkers are crucial tools for early detection and prevention, offering insights into the molecular and physiological changes preceding the onset of obesity‐related diseases. Biomarkers such as leptin, adiponectin, and PAI‐1 enable the identification of at‐risk individuals before clinical symptoms manifest, facilitating timely intervention strategies to mitigate the progression of metabolic and cardiovascular complications [192, 193]. Circulating biomarkers, including adipokines and inflammatory markers, have shown promise for noninvasive diagnostics, enhancing monitoring capabilities and guiding early prevention efforts [194]. Furthermore, integrating multicomponent biomarkers, such as genetic predispositions and lifestyle factors, improves risk stratification and allows for tailored preventive measures [195]. By refining obesity characterization beyond traditional metrics like BMI, biomarkers enable a deeper understanding of the disease's pathophysiology and provide a foundation for personalized health interventions aimed at reducing long‐term risks [196]. However, the successful translation of these advancements into clinical practice requires ongoing validation to ensure their efficacy and applicability across diverse populations.

### 12.2 Personalized Medicine

Obesity biomarkers play a pivotal role in advancing personalized medicine by enabling tailored interventions based on individual biological profiles. Biomarkers like leptin, adiponectin, and CRP can identify nonresponders to treatments, high‐risk patients, and specific obesity‐related complications, guiding individualized therapeutic strategies [192]. These biomarkers facilitate refining diagnoses beyond traditional metrics, offering insights into chronic disease risks and enabling targeted prevention measures [196]. For instance, inflammatory and DNA damage markers can help customize post‐bariatric surgery care, improving outcomes in metabolic syndrome and other obesity‐related diseases. Similarly, epigenetic and microbiota‐related biomarkers support behavior‐change strategies and treatment customization, enhancing the efficacy of obesity management approaches [197]. With advancements in multiplexing and bioassay technologies, biomarkers now show potential for personalizing obesity interventions across clinical and surgical contexts [198]. These innovations underline the critical role of biomarkers in tailoring medical care, improving health outcomes, and addressing obesity's multifaceted challenges.

### 12.3 Predicating Treatment Response

Obesity biomarkers provide valuable insights into predicting treatment responses, enabling the development of personalized therapeutic approaches. Leptin and adiponectin, as well as inflammatory markers like cytokines, have been shown to predict treatment outcomes, such as weight regain or dropouts in pediatric and adult populations, highlighting the need for targeted intervention strategies [193]. Biomarkers like caffeine metabolism–related metabolites and fasting plasma ghrelin levels have been identified as predictive of greater weight loss success in lifestyle interventions and pharmacotherapy, respectively, with fasting plasma ghrelin levels achieving a predictive accuracy of 86% for liraglutide therapy response [199].

Emerging molecular markers such as miRNAs are gaining attention for their diagnostic and predictive potential. For instance, miR‐378 and miR‐142 levels in adipose tissue and plasma correlate with obesity stages and treatment responses, with miR‐378 increasing in early obesity stages and miR‐142 decreasing as obesity progresses. These miRNAs could serve as biomarkers for the effectiveness of interventions like sibutramine therapy. Additionally, integrating genetic and metabolic profiles with clinical data, as demonstrated in bariatric and metabolic surgery research, offers a more nuanced understanding of individual variability in treatment outcomes. Preoperative biomarkers, such as those linked to adiposity and insulin resistance, can identify nonresponders and guide surgical approaches, while postoperative behavior remains a significant determinant of long‐term success [200].

The combination of these findings highlights the importance of adopting a systems approach to obesity management, leveraging biomarker data to predict treatment responses more accurately and tailoring interventions to individual patient profiles. This approach promises improved adherence, efficacy, and long‐term outcomes in obesity management.

### 12.4 Monitoring Disease Progression

Obesity biomarkers play a crucial role in monitoring disease progression, offering insights into pathophysiological pathways and enabling personalized treatment strategies. Circulating markers like adipokines, cytokines, and proteomic signatures are valuable tools for assessing obesity‐related complications and tracking metabolic alterations over time [193]. Cell‐free RNA (cfRNA) and epigenetic biomarkers further enhance prognostic capabilities, identifying individuals with favorable outcomes postintervention and providing a deeper understanding of disease mechanisms [197]. ML applications have advanced biomarker analysis, allowing complex data integration to predict outcomes and stratify patients for precision treatment [201].

Additionally, obesity‐related inflammatory markers and omics‐derived biomarkers offer potential for tracking therapeutic efficacy and disease progression, facilitating targeted interventions and weight management strategies [202]. Biomarkers like adiponectin, which increases postweight loss, highlight physiological changes associated with successful interventions [203]. However, further validation of novel biomarkers is needed to improve their reliability in clinical applications [198]. Collectively, these biomarkers offer a dynamic approach to understanding obesity and managing its associated risks effectively.

## 13 Challenges in Clinical Implementation

While obesity biomarkers hold great promise for monitoring disease progression, their clinical implementation faces significant challenges. High variability in biomarker levels due to individual metabolic differences, lack of standardization in assay methods, and limited reproducibility across studies complicate their use in routine practice [193, 198]. Further, many biomarkers, such as resistin and adiponectin, have dual roles, making their interpretation in clinical contexts complex and necessitating additional validation studies [203]. The integration of advanced technologies like imaging and ML shows potential for improved sensitivity and precision, but these approaches require robust validation and infrastructure, which are often lacking in clinical settings [201]. Moreover, challenges arise from the need to account for behavioral and lifestyle parameters, as biomarkers alone may not fully capture obesity's multifaceted etiology [197]. A consistent framework for noninvasive tests, larger cohort studies, and comprehensive multi‐omics approaches is essential to overcome these barriers and enhance the reliability and applicability of biomarkers in obesity management [204, 205]

## 14 Conclusions

Obesity has become a major public health concern on a global scale, with different regions facing unique challenges. The obesity epidemic is driven by several key factors, including urbanization, migration to urban areas, sedentary lifestyles, insufficient physical activity, and the widespread consumption of energy‐dense diets. Emerging research has identified various potential biomarkers—such as miRNAs, adipocytes, oxidative stress markers, and the microbiome—that show promise in the identification and understanding of obesity. Given the complex and interconnected nature of obesity and its effects, a comprehensive and multifaceted preventive strategy is essential. However, translating biomarker research into clinical practice presents challenges. High costs, limited accessibility, and variability in standardization can hinder widespread adoption. Addressing these issues involves developing cost‐effective assays, enhancing healthcare infrastructure, and establishing standardized testing protocols. Regulatory considerations are also crucial; biomarkers must meet stringent standards of analytical validity, clinical validity, and utility to gain approval. Collaborative efforts among researchers, clinicians, and regulatory bodies are vital to streamline the approval process and integrate biomarkers into routine obesity management. Future studies should prioritize the investigation of the sensitivity and specificity of anthropometric assessment tools in relation to these promising biomarkers. Such research could lead to significant cost savings in healthcare by enabling the early detection of obesity, thus facilitating timely intervention and management. By addressing these challenges and adopting a proactive, evidence‐based approach, we can more effectively combat the obesity epidemic and mitigate its associated health consequences.

## Author Contributions

A.A. drafted the manuscript. D.K. and M.K. designed the study, drafted the manuscript, generated the figures and tables, and reviewed the manuscript. A.K. helped working with figures. E.O. reviewed the manuscript. K.C. helped in resources. C.B. reviewed the manuscript. C.P. helped working with figures and reviewed the manuscript. F.O. helped working with figures and reviewed the manuscript. All authors have endorsed the final manuscript.

## Ethics Statement

The authors have nothing to report.

## Conflicts of Interest

The authors declare no conflicts of interest.

## Data Availability

Data available on request from the authors.
